# Hydrated Metal Vanadate Heterostructures as Cathode Materials for Stable Aqueous Zinc-Ion Batteries

**DOI:** 10.3390/molecules29163848

**Published:** 2024-08-14

**Authors:** Siqi Zhang, Yan Wang, Yunyu Wu, Guanlun Zhang, Yanli Chen, Fengyou Wang, Lin Fan, Lili Yang, Qiong Wu

**Affiliations:** Key Laboratory of Functional Materials Physics and Chemistry (Ministry of Education), College of Physics, Jilin Normal University, Changchun 130103, China; 13596803100@163.com (S.Z.); 17845068779@163.com (Y.W.); 18977966327@163.com (Y.W.); z202415204542462@163.com (G.Z.); ylchen@jlnu.edu.cn (Y.C.); wfy@jlnu.edu.cn (F.W.); fanlin@jlnu.edu.cn (L.F.)

**Keywords:** cation pre-intercalation, layered hydrated vanadate, heterostructures, aqueous zinc-ion battery

## Abstract

Aqueous zinc ion batteries (AZIBs) have received a lot of attention in electrochemical energy storage systems for their low cost, environmental compatibility, and good safety. However, cathode materials still face poor material stability and conductivity, which cause poor reversibility and poor rate performance in AZIBs. Herein, a heterogeneous structure combined with cation pre-intercalation strategies was used to prepare a novel CaV_6_O_16_·3H_2_O@Ni_0.24_V_2_O_5_·nH_2_O material (CaNiVO) for high-performance Zn storage. Excellent energy storage performance was achieved via the wide interlayer conductive network originating from the interlayer-embedded metal ions and heterointerfaces of the two-phase CaNiVO. Furthermore, this unique structure further showed excellent structural stability and led to fast electron/ion transport dynamics. Benefiting from the heterogeneous structure and cation pre-intercalation strategies, the CaNiVO electrodes showed an impressive specific capacity of 334.7 mAh g^−1^ at 0.1 A g^−1^ and a rate performance of 110.3 mAh g^−1^ at 2 A g^−1^. Therefore, this paper provides a feasible strategy for designing and optimizing cathode materials with superior Zn ion storage performance.

## 1. Introduction

The worldwide environmental degradation and energy deficit have inspired the pursuit of safe and advanced energy storage (AES) technologies [1,2]. Among the known AES technologies, aqueous electrolyte-based batteries containing multivalent (Zn^2+^, Ca^2+^, Mg^2+^, and Al^3+^) or monovalent (Li^+^, Na^+^, and K^+^) cations as the working ion have received exceptional amounts of interest, introducing outstanding advantages, for example good safety features, high ionic conductivity, environmentally friendliness, and cost effectiveness [3,4]. In particular, aqueous zinc-ion batteries (AZIBs) are attractive, with water-stable Zn as the anode, primarily because of their intrinsic safety, ease of manufacture, high redox potential (−0.78 V), and high capacity (820 mAh g^−1^) [5,6]. Unfortunately, the inferior capacities and unsatisfactory long-term cyclability of AZIBs are exacerbated by the low reversibility and structural instability of the cathode materials [7,8]. Hence, the search for high-capacity and stable-structure cathode materials can lead to the requirements for high capacities and long cycle performances in AZIBs to be met.

Researchers have conducted many studies on the various types of cathode materials, for example vanadium-based oxides, manganese-based oxides, organics/polymers, and Prussian blue analog materials [9,10,11,12]. Among them, vanadium-based oxides materials (such as Zn_0.25_V_2_O_5_ [13], NaCa_0.6_V_6_O_16_·3H_2_O [14], and MnV_12_O_31_·10H_2_O [15]) have received much attention owing to the multiple valence states of their V element and their adjustable ion transfer channels [16,17,18,19]. Specifically, their layered structure, composed of VO_x_ layers, makes the embedding and de-embedding of Zn^2+^ relatively easy. Meanwhile, they contain mixed valence states, and the higher average valence of V causes them to have more active sites, which allows for stronger redox reactions and higher capacity [20,21]. Impressively, Li et al. prepared hydrated porous δ-Ni_0.25_V_2_O_5_·nH_2_O cathode materials with a reversible capacity of 402 mAh g^−1^ at 0.2 A g^−1^ and a capacity loss of only 2% at 5 A g^−1^ after 1200 cycles [22]. Moreover, polyvanadate-Na6V_10_O_28_ with (V_10_O_28_)^6−^ an anion was provided by Zhou et al. as an aqueous ZIB cathode. Due to the high stability of its (V_10_O_28_)^6−^ clusters, the material could withstand the reversible (de)insertion of Zn^2+^, and its cycle life was effectively enhanced [23]. However, the VO_x_ layers in the layered structure were connected by hydrogen bonds, resulting in a strong electrostatic interaction between the host lattice, vanadium, which hindered the insertion of Zn^2+^ and reduced the diffusion rate [24,25]. In view of the existing problems, one of the effective strategies is to pre-intercalate new metal cations (such as K^+^, Na^+^, Ni^2+^, Ca^2+^, and Mg^2+^) into VO_x_ layers, which can enlarge interplanar spacing and improve the ion diffusion rate and electronic conductivity [26,27]. However, from the viewpoint of synthesis, the introduction of metal ions does not completely solve the problem of low diffusion rates [28,29]. Compared with pure substances, well-designed composite materials allow for faster ion/electron transfer due to the synergistic effect between two or more components [30,31]. More significantly, heterogeneous structures with rich interfaces effectively improve Zn ion transfer channels and therefore electrical conductivity [32,33]. Shan et al. prepared the biphasic vanadate Na_1.2_V_3_O_8_/K_2_V_6_O_16_·1.5H_2_O and demonstrated the benefits of the novel interface. It showed an excellent capacity of 267 mAh g^−1^ at 5 A g^−1^ after 800 cycles. The proposed novel interfacial adsorption–insertion mechanism facilitates the analysis of the performance enhancement mechanism of biphasic and multiphasic materials [34]. Therefore, an effective strategy combining a heterogeneous structure and cationic pre-intercalation is of important value for the design and optimization of high-performance AZIB cathodes.

Herein, a novel CaV_6_O_16_·3H_2_O@Ni_0.24_V_2_O_5_·nH_2_O structure (marked as CaNiVO) was prepared by combining a heterogeneous structure with cation pre-intercalation strategies as a high-performance cathode material for AZIBs. Combining electrochemical characterization and kinetic analysis, we show that a heterogeneous structure combined with cation pre-intercalation strategies promotes the electrochemical performance of CaNiVO. In addition, the introduction of Ca/Ni ions can expand the interlaminar structure and maintain the structural stability of materials. Meanwhile, the rational constructions of heterointerfaces can effectively promote electron/ion transport dynamics and establish an excellent interlayer conductive network. As we expected, the CaNiVO cathode materials showed excellent capacity (334.7 mAh g^−1^ at 0.1 A g^−1^) and a specific rate performance of 110.3 mAh g^−1^ at 2 A g^−1^. Meanwhile, the CaNiVO cathode materials achieved a long cycle performance of 82.6 mAh g^−1^ (over 500 cycles at 2 A g^−1^), and showed 83% capacity retention. This strategy can effectively optimize the overall performance of AZIBs and facilitate their further development in practical applications.

## 2. Results and Discussion

**Figure 1:**
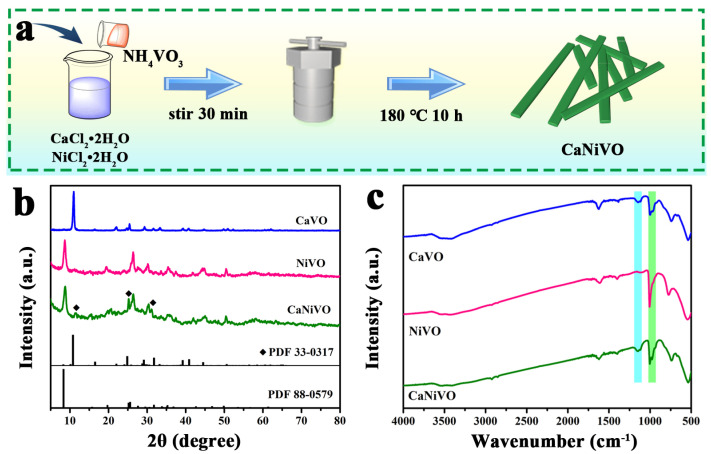
(**a**) Synthetic scheme of CaNiVO composites; (**b**) XRD patterns of CaVO, NiVO, CaNiVO, and standard phase; (**c**) FTIR spectra of CaVO, NiVO, and CaNiVO.

The synthesis procedure used for the CaNiVO heterostructure composites is schematically presented in Figure 1a. NiCl_2_·2H_2_O, CaCl_2_·2H_2_O, sodium dodecyl sulfate (SDS), and NH_4_VO_3_ were configured into the mixed solution using deionized water, and then CaNiVO was obtained via the facile one-step hydrothermal method. The phase structure of the prepared CaVO, NiVO, and CaNiVO were analyzed via X-ray diffraction (XRD) tests (Figure 1b). The XRD pattern of CaVO and NiVO as the control samples was revealed via single-interlayer cation doping, leading to the indexing of CaV_6_O_16_·3H_2_O (PDF No. 33-0317) and Ni_0.24_V_2_O_5_·nH_2_O (PDF No. 88-0579). The (001) peaks for CaVO and NiVO exhibit the strongest intensity, confirming the high crystallinity of the compounds. The structural configurations of CaVO and NiVO are exhibited in a diagram (Figure S1). Among them, the crystal structure of CaV_6_O_16_·3H_2_O consisted of a layered structure with VO_5_ square pyramids and VO_6_ octahedrons, and with calcium ions and crystal water embedded in the interlayer [35]. For Ni_0.24_V_2_O_5_·nH_2_O, crystal water and Ni ions served as pillars in the VOx layers’ structure and formed a stable tunneling structure [22]. Meanwhile, the interlayer spacing of VOx layers from NiVO was determined to be about ~10.38 Å, which is larger than that of CaVO (~8.10 Å) [36,37]. Subsequently, the characteristic peaks of CaVO and NiVO could be simultaneously observed in the CaNiVO materials, suggesting that the pre-intercalation of two metals did not affect their own structures. This also proves that CaNiVO is mainly a two-phase coexistence composite. Meanwhile, the interlayer distance values for the main peak in the XRD spectra of CaNiVO was determined to be about ~10.02 Å. Therefore, the heterogeneous structure consists of adjacent VO_x_ layers, and the interlayer space is occupied by Ni-ion or Ca-ion complexes and water molecules serving as pillars, which facilitates the shuttling of Zn^2+^ and rapid charge migration. For a further investigation of the structural information, FTIR spectra analysis was employed. With the insertion of Ca ion or Ni ion, CaNiVO, Ca_5_Ni_1_VO, and Ca_1_Ni_5_VO showed structures similar to CaVO and NiVO, which agreed with the FTIR results. Figure 1c exhibits the peaks of the V-O-V bonds from the symmetric stretching vibration (776 and 560 cm^−1^), and those of the V=O bonds from the stretching vibrations (1006 cm^−1^) [38]. Meanwhile, compared with that in CaVO and NiVO, the detected peak in CaNiVO at 738 cm^−1^ showed a significant shift, which may have been caused by the interlayers with both Ca and Ni ions embedded [39]. The broad peaks located near 1620 cm^−1^ and 3423 cm^−1^ corresponded to the O-H vibrations from structural water [40]. The weak peak at 1148 cm^−1^ was detected simultaneously in CaVO, CaNiVO, Ca_5_Ni_1_VO, and Ca_1_Ni_5_VO samples and may have corresponded to the Ca-O vibration mode. Meanwhile, the intensity of the characteristic peaks of Ca-O increased with the increase in CaV_6_O_16_·3H_2_O in different samples (Figure S2). The above results of the tests further prove the successful synthesis of CaNiVO.

**Figure 2:**
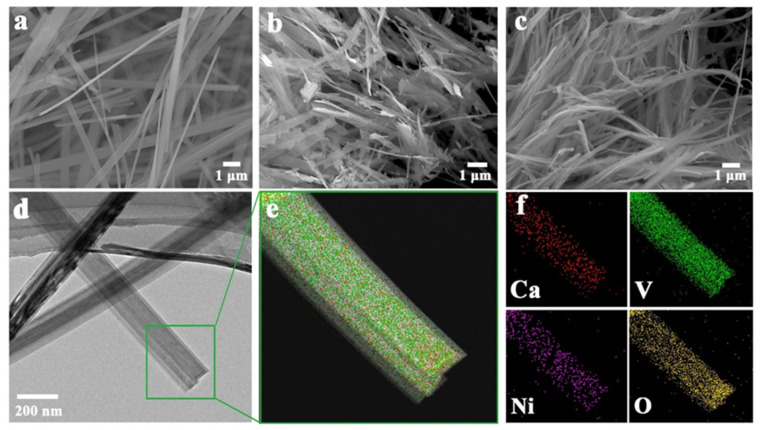
SEM images of (**a**) CaVO, (**b**) NiVO, and (**c**) CaNiVO; (**d**) TEM image of CaNiVO; and (**e**,**f**) corresponding elemental mapping results of CaNiVO.

To characterize the morphologies and structures of CaVO, NiVO, and CaNiVO composites, scanning electronic microscopy (SEM) and transmission electron microscopy (TEM) were performed. SEM images of CaNiVO mainly showed a belt-like morphology similar to that of CaVO and NiVO (Figure 2a–c). The nanoribbon structure measuring tens of microns showed good homogeneity. Meanwhile, TEM images showed a relatively high aspect ratio with the length of the nanoribbons measuring tens of micrometers and the width measuring ≈ 200 nm (Figure 2d). The high-resolution TEM (HRTEM) image showed defined lattice fringes of 0.347 nm and 0.358 nm, originating from the NiVO (110) phase and CaVO (300) phase (Figure S3). Additionally, the CaNiVO heterostructure composites formed after the intercalation of Ca ions and Ni ions were further evidenced by the elemental mapping results (Figure 2e,f), which showed that Ni, C, O, Co, and V were uniformly homogeneously distributed throughout the entire nanoribbon. Meanwhile, the atomic ratio of Ni, Ca, and V in CaNiVO was 0.32: 0.27:1 based on the energy-dispersive spectrometer (EDS) results (Figure S4). A thermo-gravimetric (TG) analysis of CaNiVO under atmospheric air was employed (Figure S5). The weight loss before heating to 100 °C can be ascribed to the evaporation of physically adsorbed water. Then, the weight loss was ascribed to the loss of 17.8% of the crystal structure in the water between 100 and 550 °C [41]. As the temperature continued to rise, there was a slow weight gain, which can be attributed to the oxidation of the pre-intercalated metal ions. The specific surface area of CaNiVO was further determined through the nitrogen adsorption–desorption isotherms (Figure S6). CaNiVO had a specific surface area of 254.69 m^2^/g, which is conducive to the wetting and penetration of electrolytes and provides more channels for Zn^2+^ insertion/extraction, further optimizing the superior reaction kinetics. Therefore, the combination of test results suggests that the CaNiVO-heterostructure composites were successfully synthesized here.

**Figure 3:**
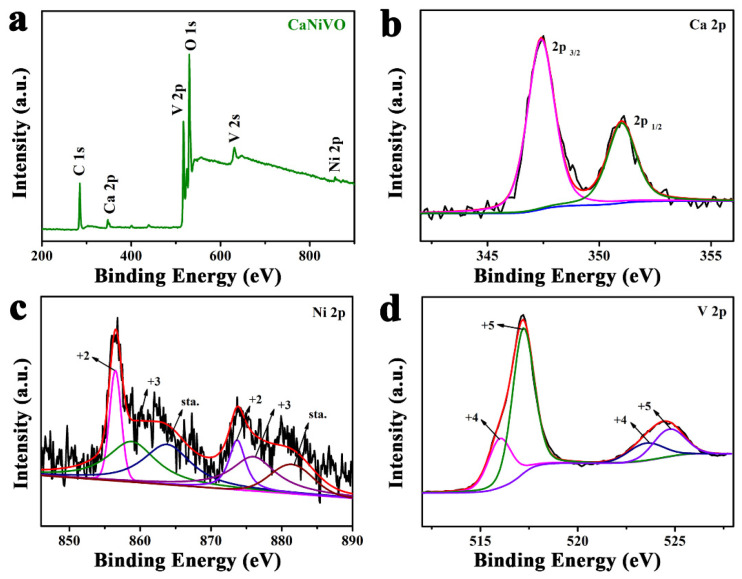
(**a**) Full-survey XPS spectrum of CaNiVO. High-resolution XPS spectra of (**b**) Ca 2p, (**c**) Ni 2p, and (**d**) V 2p of CaNiVO.

To further confirm the valence states of various elements, Figure 3a shows the results of X-ray photoelectron spectroscopy (XPS) conducted on CaNiVO. The peaks of the Ca, Ni, V, and O elements obviously appear on the XPS full spectrum, and are largely in agreement with the EDS and TEM results. For the Ca 2p spectrum, the two peaks corresponded to Ca 2p_1/2_ and Ca 2p_3/2_ at 351 and 347.4 eV, respectively (Figure 3b). In the narrow scan of Ni 2p, the six components’ peaks were found to be located at 856.5/858.8/863.8 eV and 873.7/876/881.4 eV, as shown in Figure 3c, which correspond to the two chemical states of Ni^2+^ and Ni^3+^, respectively [42]. In the V 2p spectra, the peaks for V 2p_1/2_ (523.5 eV) and V 2p_3/2_ (516 eV), corresponded to the valence state of V^4+^ (Figure 3d). The peaks of 517.2 and 530.2eV, corresponding to V 2p_3/2_ and V 2p_1/2_, were rooted in V^5+^ [43,44]. Furthermore, the O 1s spectrum shows peaks at 529.9, 531, and 532.7 eV, which can be assigned to O^2−^ and OH^−^ and H_2_O, respectively [45] (Figure S7).

**Figure 4:**
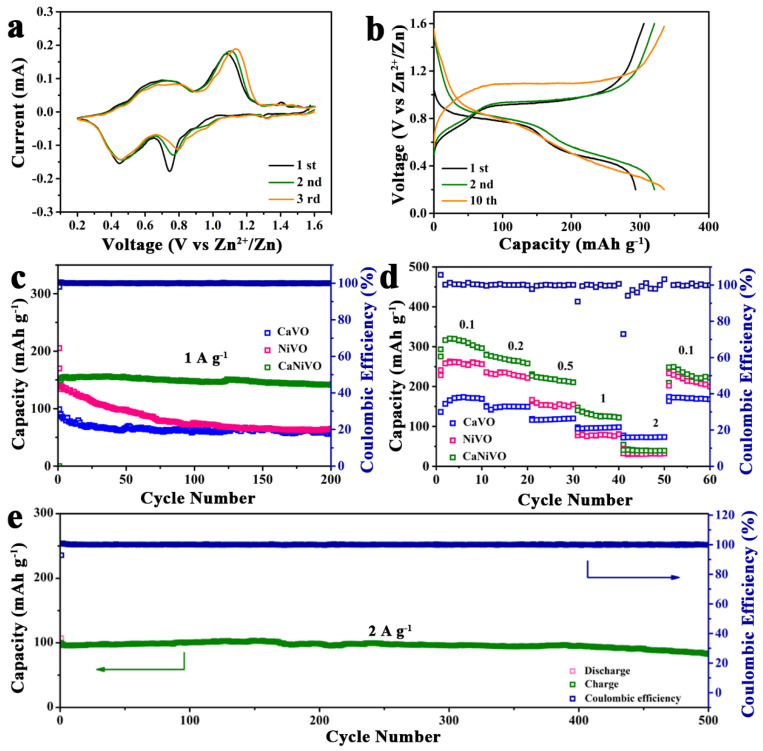
(**a**) CV curves of CaNiVO at 0.1 mV s^−1^, (**b**) discharge/charge profiles of CaNiVO at 0.1 A g^−1^, (**c**) cycling performance of various samples at 1 A g^−1^, (**d**) rate capability of CaNiVO, and (**e**) long cycling stability evaluation of CaNiVO at 2 A g^−1^.

To investigate the impacts of the heterogeneous structure generated via Ca/Ni preintercalation on the energy storage properties, AZIBs were assembled in coin cells with metallic Zn foil as the anode, 3 M Zn(CF_3_SO_3_)_2_ aqueous solution as the electrolyte, and CaNiVO as the cathode. In Figure 4a, the initial three CV cycles of CaNiVO are shown at a voltage range of 0.2–1.6 V with a scan rate of 0.1 mV s^−1^. The CV curves in the initial scan show two pairs of redox peaks, implying a multi-step intercalation/deintercalation of the Zn^2+^ process. The initial scan showed cathodic peaks at 0.74 and 0.44 V, corresponding to the stepwise reduction reaction from V^5+^ to V^4+^ and V^3+^, a process caused by Zn^2+^ insertion into CaNiVO [46]. Meanwhile, the following anodic peaks at 0.68 and 1.08 V can be ascribed to the extraction of Zn^2+^ from CaNiVO [47]. Note that the subsequent cycles of CaNiVO almost overlap with other cycles, showing the reversible (de) intercalation of Zn^2+^ behavior. The galvanostatic charge and discharge (GCD) profiles of the voltage plateaus are consistent with the multi-step (de)intercalation of Zn^2+^ in the CV curves (Figure 4b and Figure S8). In the GCD profiles of CaNiVO at 0.1 A g^−1^ in the different numbers of cycles, the first specific discharge/charge capacities reached 293.6/305.6 mAh g^−1^, which is significantly higher than that of CaVO of 135.9/136.4 mAh g^−1^ and that of NiVO of 229.1/241.1 mAh g^−1^. Meanwhile, all the materials showed that the second cycle’s capacity was higher than the first cycle’s capacity, due to the gradual activation of the material, and a similar result was observed in previous work on vanadate as an electrode material for AZIBs [48,49]. For the 10th cycle, CaNiVO, CaVO, and NiVO showed reversible discharge/charge capacities of 334.7/337.1 mAh g^−1^, 168.6/168.6 mAh g^−1^, and 256.2/256.6 mAh g^−1^, corresponding the coulombic efficiency (CE) values of 100.7%, 100%, and 100.1%, respectively, which demonstrate good electrochemical reversibility. Subsequently, the long-term cycling performance can be observed at 1 A g^−1^ from Figure 4c. CaNiVO had the highest initial discharge/charge capacities compared with CaVO and NiVO. Furthermore, CaVO after 200 cycles maintained a capacity of 141.1 mAh g^−1^ (CaVO had a capacity of 57.9 and NiVO of 64.7 mAh g^−1^), demonstrating prominent high electrochemical reversibility. To determine the cause of this excellent cyclic stability, SEM images of CaVO after 200 cycles were tested (Figure S9). The nanoribbon structure of the CaVO and the super P-conductive additives and PVDF added during electrode preparation can be observed. The excellent structural stability of CaVO is due to the metal ions embedded between the layers and the heterogeneous interface of the two phases modulating the volume changes during the cycling. The rate performance of various samples can be seen in Figure 4d. The specific capacities of CaNiVO were 313.8, 274.6, 218, 173.6, and 110.3 mAh g^−1^ at the current densities of 0.1 to 2 A g^−1^. When the current density returned to 0.1 A g^−1^, the specific capacity was still 278.3 mAh g^−1^, which is higher than that of CaVO (174.1 mAh g^−1^) and NiVO (201.8 mAh g^−1^). Meanwhile, the corresponding GCD profiles of CaNiVO show similar voltage platforms, confirming the reversibility of the electrochemical reaction and fast charge transfer kinetics even at a high current density (Figure S10). More importantly, compared with Ca_5_Ni_1_VO and Ca_1_Ni_5_VO, CaNiVO showed stable cycle performance after 500 cycles with a capacity for retention of 83% even at the current density of 2 A g^−1^ (Figure 4e and Figure S11). The results clearly show that the heterogeneous structure caused by the pre-intercalation of cations reduced the electrostatic attraction of Zn^2+^ in the layer of vanadate, accelerated the migration rate of Zn^2+^, and led to remarkable rate performance. The stability of the interlayer structure, on account of the pillars of calcium ions and nickel ions, was maintained, which reduced vanadium dissolution. To qualitative analyze the V-dissolution, optical images of the separator at 2 A g^−1^ after 200 cycles of CaNiVO are provided (Figure S12). The separator did not show significant discoloration. Subsequently, results of the open circuit potential (OCP) resting test conducted at 1 A g^−1^ for 2 days were provided for the quantitative analysis of V-dissolution. Both structural deformation and V-dissolution rates affected the recharge capacity, causing it to lower (Figure S13) [50]. Neglecting small structural effects, the extent of recharge capacity loss can be considered approximately equal to that of vanadium dissolution. The dissolution of CaVO, NiVO, and CaNiVO occurred at 13.3%, 12.8% and 10.6%.

**Figure 5:**
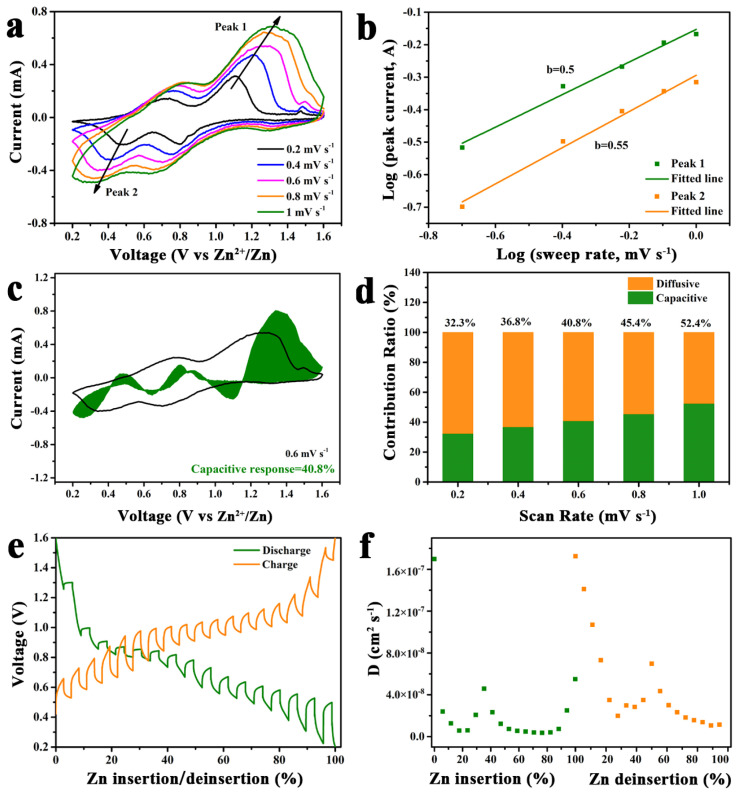
(**a**) CV curves at different scan rates for CaNiVO within 0.2–1 mV s^−1^; (**b**) relationship between peak currents and scan rates; (**c**) contribution ratio at a scan rate of 0.6 mV s^−1^; (**d**) capacitive contribution of CaNiVO at various scan rates; (**e**) GITT plot and (**f**) corresponding Zn^2+^ diffusion coefficient of CaNiVO.

Figure 5a displays the CV curves at various scan rates, which can allow us to further analyze the electrochemical kinetics of CaNiVO. The five CV profiles all show similar shapes. Noticeably, the current values gradually increase, and the reduction/oxidation peak goes through a slight shift with the increase in sweep rate, indicating polarization extension. The shapes of the five CV curves are similar. Noticeably, with the increase in the scanning rate, the current value gradually increases, and the reduction/oxidation peak goes through a slight shift, indicating polarization expansion. Based on the power–law relationship in i = aν^b^, the b values are determined to be 0.5/0.55 of peak 1/peak 2, respectively (Figure 5b). Meanwhile, the b values within were 0.5 and 1, meaning that the electrode reaction was a mixed mechanism of diffusion-controlled processes and the pseudocapacitive behavior of Zn^2+^ storage [51]. Subsequently, pseudocapacitive and diffusion-controlled contributions were calculated by the following equation: I = k_1_v + k_2_v_1/2_ [52]. Thus, the pseudocapacitive contribution of CaNiVO is indicated by the shaded area, which is measured to represent a contribution of 40.8% at 0.6 mV s^−1^ (Figure 5c). The pseudocapacitive contribution ratios under the scan rates from 0.2 to 1.0 mV s^−1^ are 32.3, 36.8, 40.8, 45.4, and 52.4% in Figure 5d. The capacitive contribution increased with the increasing scan rate, and it is shown that the electrochemical reaction of CaNiVO was mainly due to diffusion-controlled processes. Galvanostatic intermittent titration technique (GITT) measurements were conducted to further investigate the electrochemical kinetics. The diffusion coefficients of Zn^2+^ were calculated according to the following equation [53]:

(1) $$D_{{Zn}^{2+}}=\frac{4}{\pi \tau }{\left(\frac{m_{B}V_{M}}{M_{B}S}\right)}^{2}{\left(\frac{\Delta E_{s}}{\Delta E_{t}}\right)}^{2},$$

The diffusion coefficients of the initial cycles can be determined to be around 7.3 × 10^−10^–1.7 × 10^−7^ cm^2^ s^−1^, which is the same as that for the second cycles and indicates the fast Zn^2+^ insertion/extraction storage capability of CaNiVO (Figure 5e,f and Figure S14).

**Figure 6:**
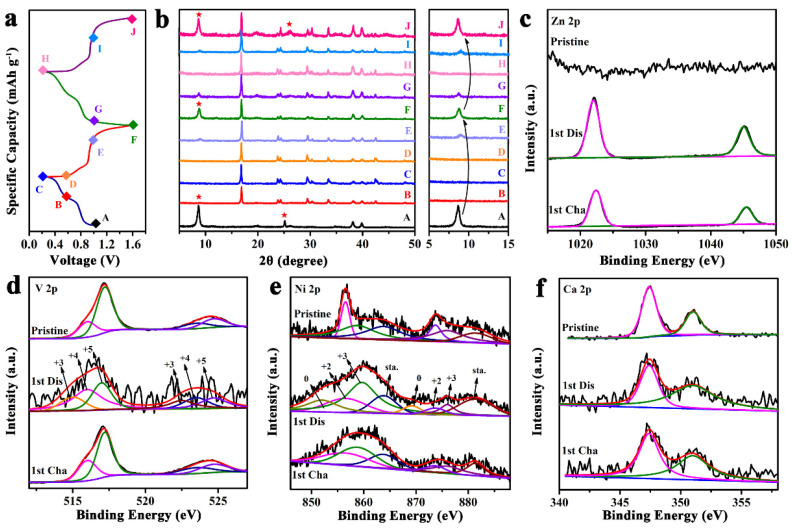
(**a**) Discharge/charge curves in the first two cycles; the matching (**b**) ex situ XRD patterns; and XPS survey spectra of (**c**) Zn 2p, (**d**) V 2p, (**e**) Ni 2p, and (**f**) Ca 2p.

To truly understand the Zn ion de/insertion mechanism of CaNiVO, ex situ XRD and XPS analyses were carried out. The ex situ XRD analyses uncovered the evolution of the crystal structure of CaNiVO in the initial two cycles during the charge/discharge processes (Figure 6a,b). When first discharged to 0.2 V, the main peaks of CaNiVO disappeared, demonstrating the amorphous transformation of the CaNiVO heterogeneous structures. Meanwhile, new phases belonging to Zn(CF_3_SO_3_)_2_ were discovered and persisted in subsequent in situ XRD tests after electrode contact with the electrolyte [54]. The ex situ FTIR spectra show similar results (Figure S15). The characteristic peaks of Zn(CF_3_SO_3_)_2_ located at 643.2 and 1033.7/1261.2 cm^−1^ correspond to the SO_3_ bending vibration and stretching vibration, and those at 1173.5/1231.4 cm^−1^ correspond to the CF_3_ stretching vibration [50]. When charged to 1.6 V, the main peak of CaNiVO was detected again, which demonstrated the reversible process of Zn^2+^ insertion/extraction. During the second cycle, the ex situ XRD pattern showed effectively the same trend, indicating the reversible electrochemical phase process of CaNiVO and its good cyclic stability. To further examine the interfacial chemistry of the electrode, ex situ XPS tests were subsequently performed during the cycling processes. As shown in Figure 6c, in the full-discharged electrode, the quite strong peaks of Zn 2p located at 1045.4 eV (Zn 2p_1/2_) and 1022.4 eV (Zn 2p_3/2_) were observed due to the successful embedding of Zn^2+^. In the fully charged state, the peak intensity decreased obviously, corresponding to the residual Zn^2+^ remaining in the structure, which is in agreement with the results of previous published studies [55]. Similarly, the peak of V^3+^ at 515 and 522.5 eV was detected in the fully discharged electrode as opposed to the pristine sample electrode, indicating the reduction of V during the intercalation of Zn^2+^ (Figure 6d). Upon charging to 1.6 V, the V^3+^ peak disappeared while the peaks of the V^4+^ and V^5+^ returned to almost the same position as that in the original states. At the same time, clear chemical state changes were detected in Ni (Figure 6e). The two peaks in the Ni spectra located at 852 and 869.2 eV correspond to Ni 0, suggesting the reduction of Ni^3+^ into Ni 0, which is in agreement with other reports of the displacement reaction mechanism. Upon charging, the peaks were recovered back to their original states, showing the reversible electrochemical storage process for CaNiVO. The shift in Ca 2p was only slight during the repeated charge/discharge processes, demonstrating that its electrochemically inert nature can ensure that Ca ions act as interlayer pillars and maintain the structural stability of VO_x_ layers (Figure 6f) [56]. The ex situ XRD, XPS, and FTIR analyses from the perspective of structural evolution indicate the stability of the VO_x_ layers and the Ca- ions/Ni ions from CaVO/NiVO, supporting the excellent electrochemical performance of CaNiVO during the de/insertion of Zn^2+^.

## 3. Materials and Methods

### 3.1. Materials

CaCl_2_·2H_2_O (Beijing Chemical Works, Beijing, China), NiCl_2_·2H_2_O (Aladdin Reagent, AR, Shanghai, China), alcohol (Beijing Chemical Works, Beijing, China), sodium dodecyl sulfate (SDS, Aladdin Reagent, AR, Shanghai, China), NH_4_VO_3_ (Aladdin Reagent, AR, Shanghai, China), polyvinylidene fluoride (PVDF, DuPont Company, Wilmington, DE, USA, 99.9%), super P-conductive additive (Hong-Xin Chemical Works, Taixing, China), N-methyl-2-pyrrolidinone (NMP, Aladdin Reagent, AR, Shanghai, China), separator (polypropylene film, Celgard 2400, Celgard, Charlotte, NC, USA), Ti foil (Chengshuo Metal Materials, Jinan, China), metallic Zn foil (Aladdin Reagent, Shanghai, China), and deionized water (Laboratory preparation) were used.

### 3.2. Material Synthesis

In the synthesis of CaNiVO, 50 mg sodium dodecyl sulfate (SDS), 150 mg NiCl_2_·2H_2_O, and 150 mg CaCl_2_·2H_2_O were added to 15 mL deionized water. Next, 58.3 mg NH_4_VO_3_ was added in 15 mL deionized water under 40 °C. After mixing the two obtained solutions, the mixture solution was moved to a Teflon-lined autoclave and maintained at 180 °C for 10 h. The resulting product, CaV_6_O_16_·3H_2_O@Ni_0.24_V_2_O_5_·nH_2_O (marked as CaNiVO), was rinsed and centrifuged with deionized water/alcohol a number of times and dried at 70 °C for 24 h. For comparison, different proportions of CaV_6_O_16_·3H_2_O and Ni_0.24_V_2_O_5_·nH_2_O were prepared, adding 250 mg CaCl_2_·2H_2_O and 50 mg NiCl_2_·2H_2_O, denoted as Ca_5_Ni_1_VO, and 50 mg CaCl_2_·2H_2_O and 250 mg NiCl_2_·2H_2_O, denoted as Ca_1_Ni_5_VO. Subsequently, pure CaV_6_O_16_·3H_2_O and Ni_0.24_V_2_O_5_·nH_2_O (denoted as CaVO and NiVO) were prepared by only adding 300 mg CaCl_2_·2H_2_O and 300 mg NiCl_2_·2H_2_O, respectively.

### 3.3. Characterizations

The microstructures of the nanomaterials were observed via field-emission SEM (JEOL, Tokyo, Japan, JSM-7800F), and TEM images were taken using a JEOL-2010 transmission electron microscope operating at a 200 kV accelerating voltage. The crystal phases were evaluated via XRD (Rigaku, Tokyo, Japan, D/max-2500PC) using Cu-Kα radiation. Thermogravimetric analysis (TG) was performed on a TA SDT 2960 simultaneous thermal analyzer at a heating rate of 10 °C min^−1^ in air. X-ray photoelectron spectroscopy (XPS) analysis was performed using an XPS system (Thermofisher, Waltham, MA, USA, Excalab 250 xi). The electrochemical performance and CV/EIS were determined using a LAND test system (CT2001A, Wuhan, China) and electrochemical workstation (PARSTAT 4000A, AMETEK Scientific Instruments, Oak Ridge, TN, USA).

### 3.4. Electrochemical Measurements

The electrodes were prepared using a typical fabrication process: 16 mg of active materials, 2 mg of polyvinylidene fluoride, and 2 mg of conductive additive (Super P) were dissolved in N-methyl-pyrrolidone solvent. After grinding for 1 h, the homogeneous slurry was casted onto Ti foil. The obtained Ti foil was cut into a circular electrode after drying at 100 °C for 12 h. Then, the obtained working electrodes were fabricated into AZIBs using the counter electrode (metallic zinc), the electrolyte (3 M Zn(CF_3_SO_3_)_2_ solution), and the separator (glass fiber membrane). The mass load of the active material at the working electrode was about 0.6~0.8 mg.

## 4. Conclusions

In summary, the heterogeneous structure CaV_6_O_16_·3H_2_O@Ni_0.24_V_2_O_5_·nH_2_O (marked as CaNiVO) was prepared using a combination of cation pre-intercalation strategies and using the simple hydrothermal method. The corresponding characterization showed that the heterogeneous structure created via the pre-intercalation of cations exhibited excellent electrochemical properties. In addition, the Ca ions and Ni ions acted as pillars between the VO_x_ layers, maximizing the expansion of the interlayer structure and reducing the electrostatic interaction during Zn^2+^ insertion/extraction from the material. Meanwhile, the heterogeneous structure of CaNiVO effectively guaranteed structural stability in the electrochemical reaction and electrochemical kinetics of the Zn^2+^ displacement reaction. Therefore, CaNiVO could offer high reversible capacity (334.7 mAh g^−1^ at 0.1 A g^−1^) and a long cycling life (with the retention of 83% of its capacity at 2 A g^−1^ after 500 cycles). This heterogeneous structure, created via pre-intercalation, may represent a new strategy to comprehensively optimize AZIB cathode materials and even other rechargeable electrode material systems.

## Data Availability

The data presented in this study are available in the article and Supplementary Materials.

## Supplementary Materials

The following supporting information can be downloaded at https://www.mdpi.com/article/10.3390/molecules29163848/s1: Figure S1: Structural configurations of (a) CaVO and (b) NiVO; Figure S2: FTIR spectra of Ca_5_Ni_1_VO and Ca_1_Ni_5_VO; Figure S3: HRTEM images of CaNiVO; Figure S4: Energy-dispersive spectrometer (EDS) results for CaNiVO; Figure S5: TG curves of CaNiVO; Figure S6: (a) Nitrogen adsorption–desorption isotherms of CaNiVO; Figure S7: High-resolution XPS spectra of O 1s; Figure S8: Charge/discharge curves of (a) CaVO and (b) NiVO; Figure S9: SEM images of CaNiVO at 1 A g^−1^ after 200 cycles at (a) low magnification and (b) high magnification.; Figure S10: Charge/discharge curves of CaNiVO at different current rates; Figure S11: Cycling performance of various samples at 2 A g^−1^; Figure S12: Optical images of V dissolution tests at 2 A g^−1^ after 200 cycles; Figure S13: Two-day OCP rest tests at 1 A g^−1^ for (a) CaVO, (b) NiVO, and (c) CaNiVO; Figure S14: (a) GITT curves and (b) corresponding Zn^2+^ diffusion coefficients at the discharge/charge state of CaNiVO in the initial cycles; Figure S15: Ex situ FTIR spectra of CaNiVO.
