# Supplementary material for: Hydrated Metal Vanadate Heterostructures as Cathode Materials for Stable Aqueous Zinc-Ion Batteries
Source: Molecules. 2024 Aug 14;29(16):3848. doi: 10.3390/molecules29163848 (PMC11357528; doi:10.3390/molecules29163848)
Supplement: Supplementary file 1 [file molecules-29-03848-s001.zip › molecules-3099697-supplementary.pdf]

Supporting Information

Hydrated Metal Vanadate Heterostructures as Cathode Materials for Stable Aqueous Zinc-Ion Batteries

Siqi Zhang, Yan Wang, Yunyu Wu, Guanlun Zhang, Yanli Chen, Fengyou Wang, Lin Fan, Lili Yang * and Qiong Wu *

Key Laboratory of Functional Materials Physics and Chemistry (Ministry of Education), College of Physics, Jilin Normal University, Changchun 130103, China; 13596803100@163.com (S.Z.); 17845068779@163.com (Y.W.); 18977966327@163.com (Y.W.); z202415204542462@163.com (G.Z.); ylchen@jlnu.edu.cn (Y.C.); wfy@jlnu.edu.cn (F.W.); fanlin@jlnu.edu.cn (L.F.)

* Correspondence: llyang1980@126.com or llyang@jlnu.edu.cn (L.Y.); qiongwu85@163.com or qiongwu@jlnu.edu.cn (Q.W.)

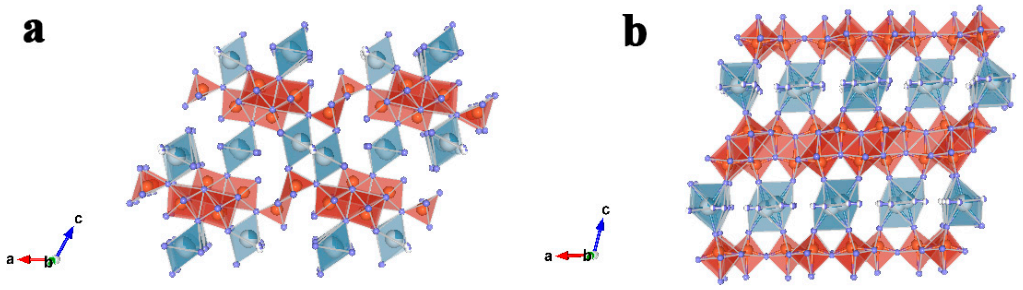

Figure S1 Structural configurations of (a) CaVO and (b) NiVO.

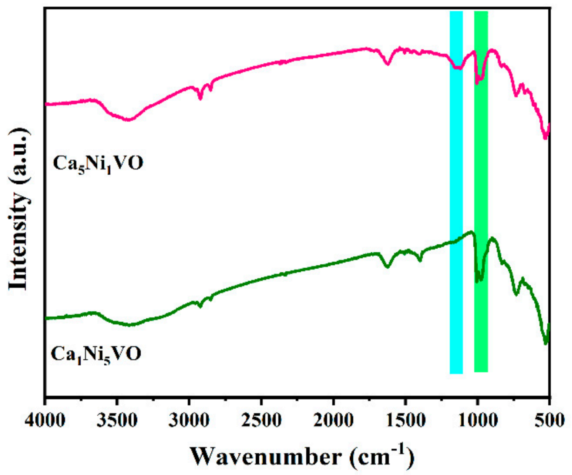

Figure S2 FTIR spectra of Ca_5Ni_1VO and Ca_1Ni_5VO.

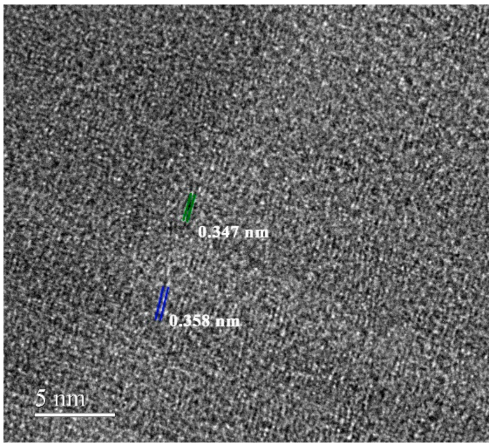

Figure S3 HRTEM images of CaNiVO.

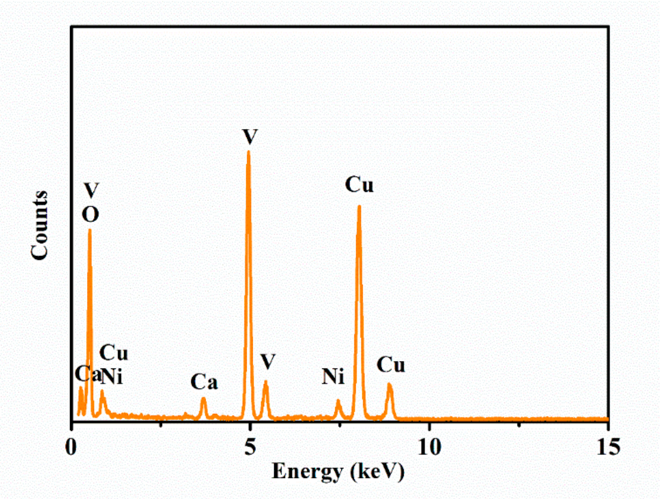

Figure S4 Energy-dispersive spectrometer (EDS) of CaNiVO.

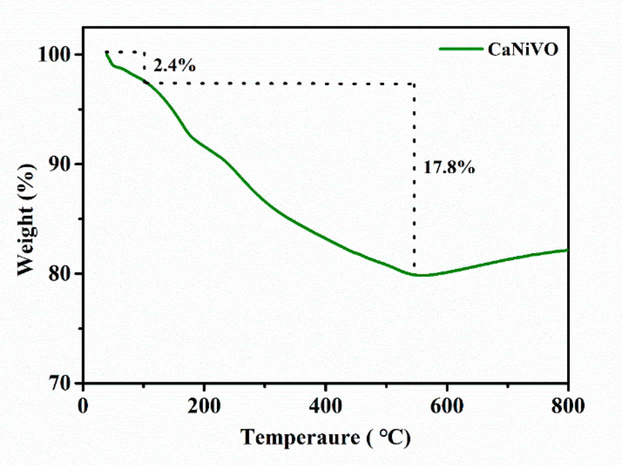

Figure S5 TG curves of CaNiVO.

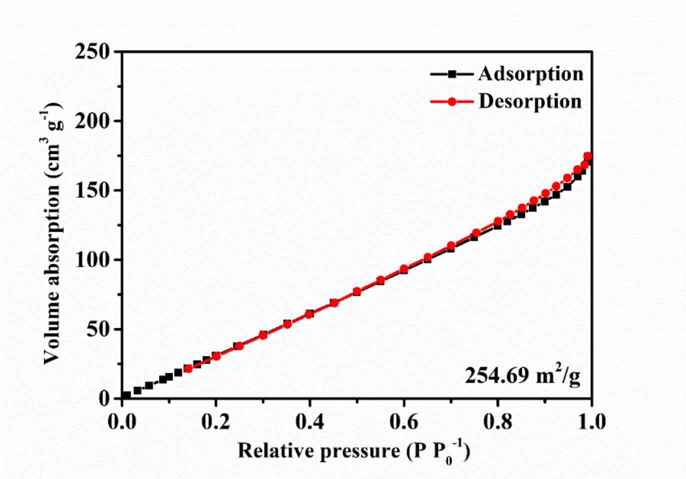

Figure S6 Nitrogen adsorption-desorption isotherms of CaNiVO.

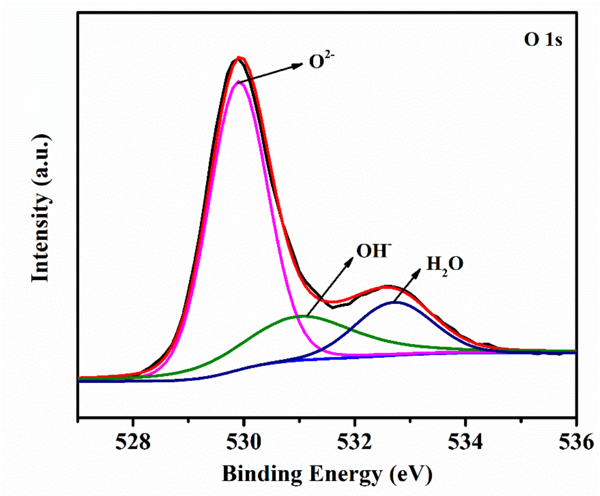

Figure S7 High-resolution XPS spectra of O 1s.

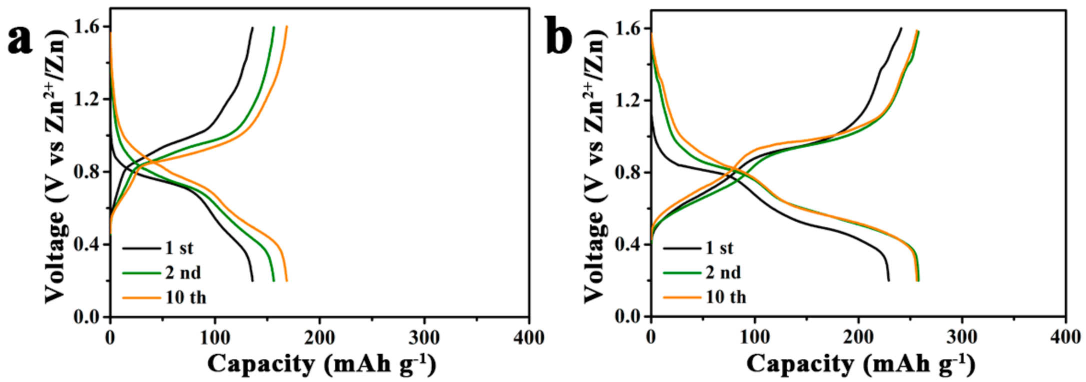

Figure S8 The charge/discharge curves of (a) CaVO, (b) NiVO.

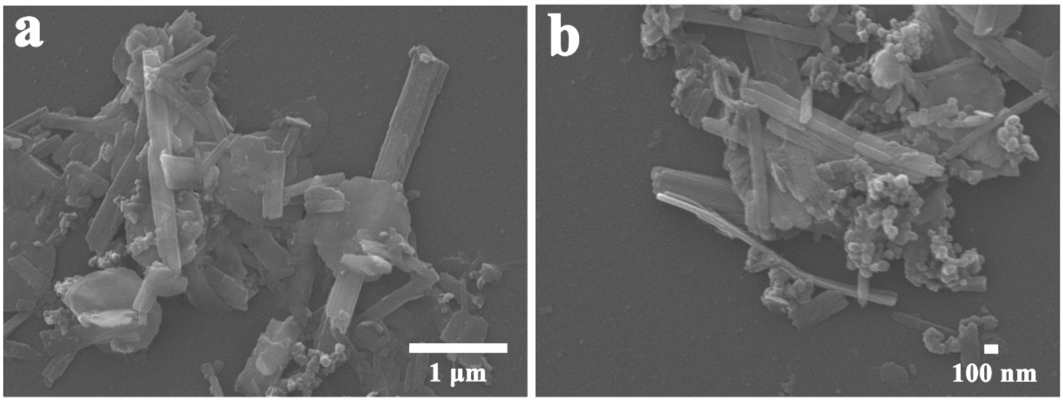

Figure S9 SEM images of CaNiVO at 1 A g^{-1} after 200 cycles at (a) low magnification and (b) high magnification.

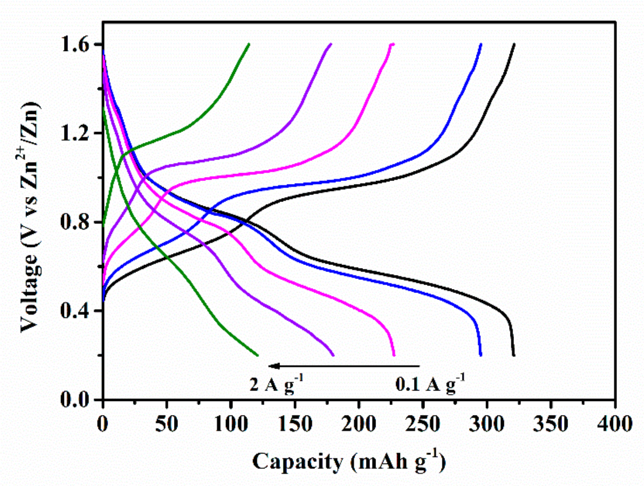

Figure S10 Charge/discharge curves of CaNiVO at different current rates.

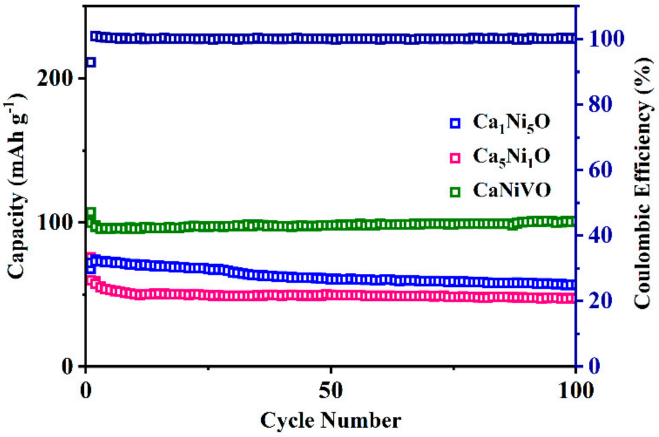

Figure S11 Cycling performance of various samples at 2 A g^{-1}.

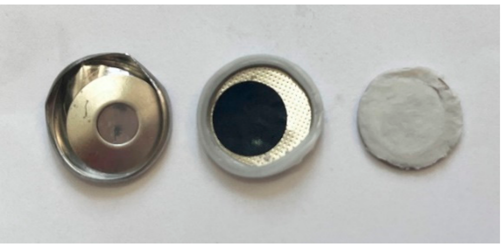

Figure S12 Optical images of V-dissolution tests at 2 A g^{-1} after 200 cycles.

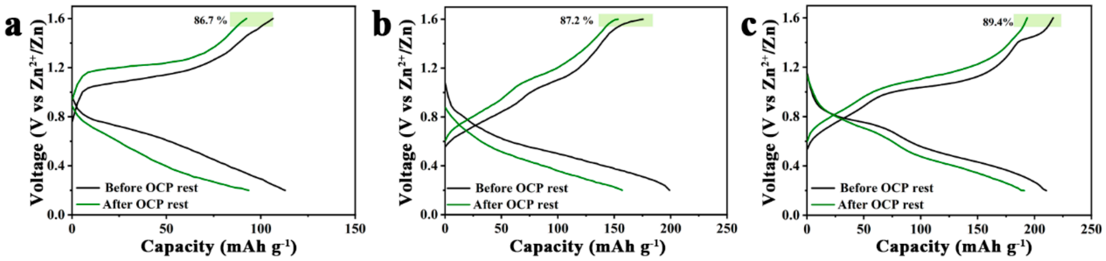

Figure S13 2-day OCP rest tests at 1 A g^{-1} of (a) CaVO, (b) NiVO and (c) CaNiVO.

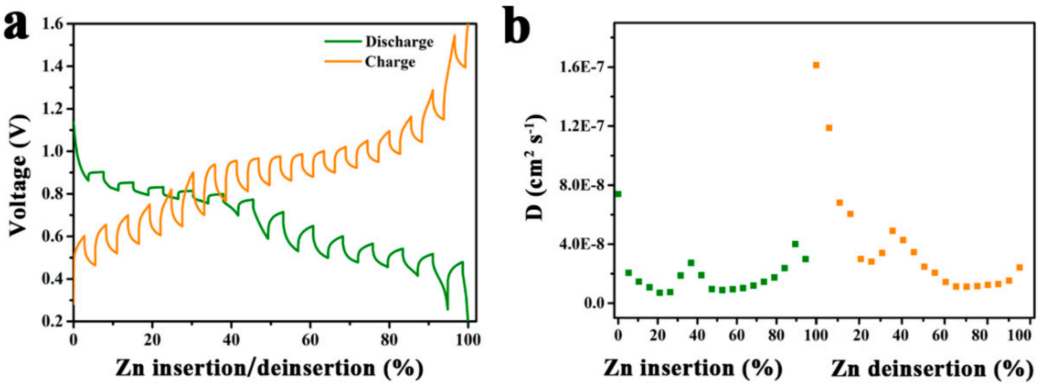

Figure S14 (a) GITT curves and (b) corresponding Zn^{2+} diffusion coefficient at discharge/charge state of CaNiVO in the initial cycles.

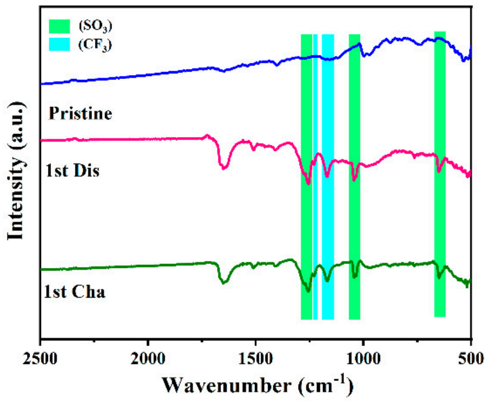

Figure S15 Ex-situ FTIR spectra of CaNiVO.
